# Supplementary material for: The Predominant Sources of Heavy Metals in Different Types of Fugitive Dust Determined by Principal Component Analysis (PCA) and Positive Matrix Factorization (PMF) Modeling in Southeast Hubei: A Typical Mining and Metallurgy Area in Central China
Source: Int J Environ Res Public Health. 2022 Oct 14;19(20):13227. doi: 10.3390/ijerph192013227 (PMC9602615; doi:10.3390/ijerph192013227)
Supplement: Supplementary file 1 [file ijerph-19-13227-s001.zip › ijerph-1944580-supplementary.pdf]

Supplementary Materials

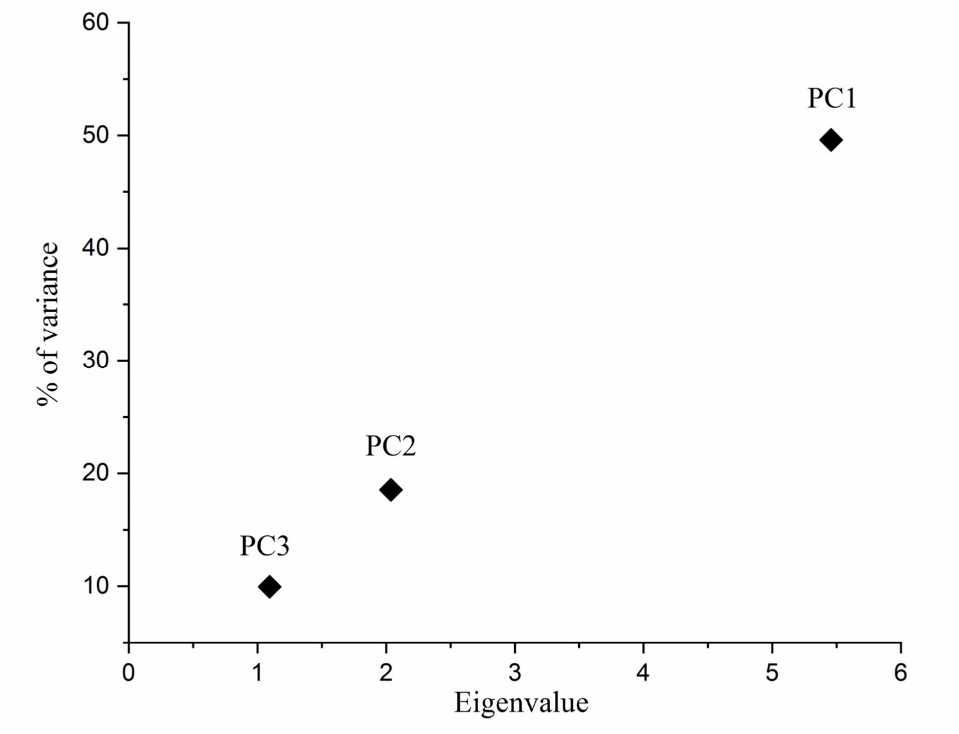

Figure S1. The varimax rotated component of PCA model

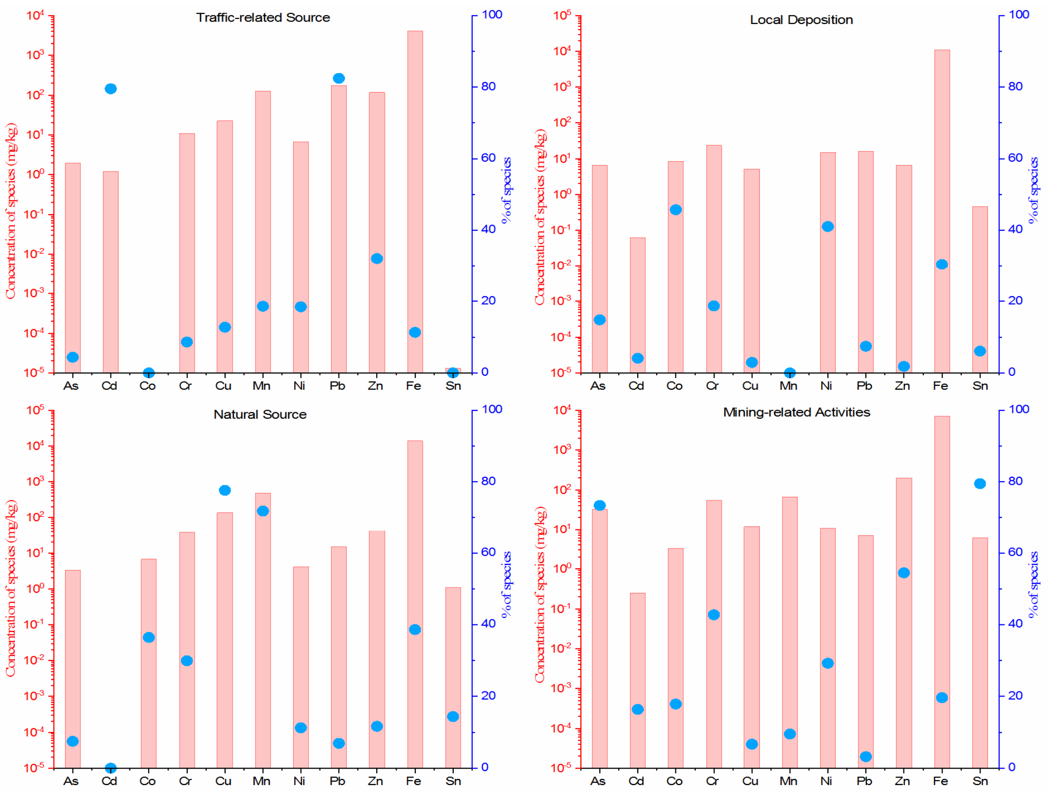

Figure S2. Factor profile of the fugitive dust from PMF. The concentration of each species apportioned to the factor as a pink bar (left y-axis) with a logarithmic scale. The percent of each species apportioned to the factor as a blue dots (right y-axis).

Table S1 The geographical coordinates for the sampling sites

ID	Type	E	N
CD-01	Construction dust	115.415249	29.853654
CD-02	Construction dust	115.184053	29.860163
CD-03	Construction dust	114.938861	30.076814
CD-04	Construction dust	114.890844	30.212297
CD-05	Construction dust	115.152771	30.203515
CD-06	Construction dust	115.054336	30.251068
CD-07	Construction dust	115.039308	30.150328
CD-08	Construction dust	114.999012	30.196967
SD-01	Soil dust	115.447570	29.843526
SD-02	Soil dust	115.229204	29.868206
SD-03	Soil dust	114.979167	30.101418
SD-04	Soil dust	114.910005	30.206190
SD-05	Soil dust	115.142567	30.199113
SD-06	Soil dust	115.046785	30.237041
SD-07	Soil dust	115.018372	30.143201
SD-08	Soil dust	115.019134	30.223766
DD-01	Deposit dust	115.435728	29.859267
DD-02	Deposit dust	115.209542	29.835639
DD-03	Deposit dust	114.965355	30.097637
DD-04	Deposit dust	114.885322	30.198385
DD-05	Deposit dust	115.111081	30.204765
DD-06	Deposit dust	115.059025	30.231526
DD-07	Deposit dust	115.045508	30.140030
DD-08	Deposit dust	115.023007	30.207474
RD-01	Road dust	115.427584	29.855425
RD-02	Road dust	115.198811	29.871793
RD-03	Road dust	114.966251	30.118253
RD-04	Road dust	114.900658	30.215673
RD-05	Road dust	115.121465	30.201323
RD-06	Road dust	115.074065	30.216039
RD-07	Road dust	115.029151	30.149268
RD-08	Road dust	115.004373	30.218175
LD-01	Landfill dust	115.431400	29.843114
LD -02	Landfill dust	115.201318	29.857343
LD -03	Landfill dust	114.950356	30.084576
LD -04	Landfill dust	115.131024	30.201207
LD -05	Landfill dust	115.069737	30.232682
LD -06	Landfill dust	115.036453	30.139763
LD -07	Landfill dust	115.017536	30.191749
LD -08	Landfill dust	114.879604	30.225914
